# Assessing the associations between public works participation, household water and sanitation conditions, and child nutrition in Southern Madagascar: a mediation analysis

**DOI:** 10.3389/fpubh.2026.1846565

**Published:** 2026-06-22

**Authors:** Fanantenana Raholiarimanana, Henintsoa Felamboahangy Rasoarahona, Solomon Bizuayehu Wassie, Akira Ishida

**Affiliations:** 1 Graduate School of Agricultural Science, Kobe University, Kobe, Japan; 2 Ecole Supérieure des Sciences Agronomiques, University of Antananarivo, Antananarivo, Madagascar; 3 Department of Agricultural Economics, Bahir Dar University, Bahir Dar, Ethiopia

**Keywords:** children, highly intensive public works (HIMO), instrumental variable, Madagascar, malnutrition, mediation, social protection, water sanitation and hygiene (WASH)

## Abstract

**Background:**

The pervasive child undernutrition in Southern Madagascar is exacerbated by inadequate water, sanitation, and hygiene (WASH) infrastructure. Although public-works programs such as high-intensity manual labor (HIMO) have been implemented to develop infrastructure and sustain livelihoods, the multi-dimensional associations between these programs and household-level health and hygiene outcomes remain poorly quantified. This study examined the association of workfare participation with child nutritional status and the role of household WASH conditions as a mediating factor.

**Methods:**

Data were integrated from the 2018 Madagascar Multiple Indicator Cluster Survey (MICS6) and 2021 Demographic and Health Survey Spatial Covariates. To address the potential endogeneity in program participation and in the WASH mediators, we employed a potential-outcomes framework combined with instrumental variable (IV) regressions. This approach allowed for the estimation of both total and mediator-adjusted associations between HIMO participation, WASH practices, and child anthropometric outcomes (*Z*-scores). Furthermore, a mediation analysis was used to quantify the proportion of this association that could be statistically explained by improvements in WASH infrastructure.

**Results:**

Participation in the HIMO program was significantly associated with higher household access to improved sanitation and with shorter water-collection travel time (under 30 min). These improvements were associated with significant increments in the height-for-age (HAZ), weight-for-age (WAZ), and weight-for-height (WHZ) *Z*-scores. Mediation analysis indicated that 12%−14% of the program's total association with the HAZ and WAZ could be statistically explained by improved sanitation infrastructure. Shorter water-fetching time accounted for the largest mediated share of the association between HIMO participation and WHZ, representing approximately 36% of the total association.

**Conclusions:**

This study provides evidence that HIMO participation is associated with better child nutrition in Southern Madagascar, with improved access to water and sanitation as potential mediating pathways. If subsequent longitudinal or experimental evidence confirms these patterns, public works could be designed as nutrition-sensitive interventions that prioritize essential infrastructure. Expanding these projects also offers a sustainable pathway to closing basic-service gaps and reducing regional malnutrition rates.

## 1 Introduction

In sub-Saharan Africa, poverty, inadequate hygiene, and disease pose major threats to life. Over 65% of the population in this region lacks access to basic sanitation services, with nearly 15% practicing open defecation (1). This deficiency in hygiene and sanitation infrastructure exposes the population to environmental enteric dysfunctions (EEDs) and infectious diseases, with children being the most susceptible (2, 3). Reliable access to improved drinking-water sources and sanitation facilities significantly enhances nutrient absorption and gut function in young children and prevents damage to the gut structure (2, 4). Diarrheal diseases among children are reduced by improved WASH practices, limiting the risk of water and mineral losses occurring during their manifestation (2, 5).

Nevertheless, many households in the Global South face substantial difficulties in accessing improved water, sanitation, and hygiene (WASH) facilities (1). Due to economic, cultural, climatic, or spatial constraints, a large proportion of the population continues to rely on unimproved sanitation facilities and lacks access to safe drinking water (6). Water scarcity is particularly acute in the arid and semi-arid regions, where limited availability and high costs restrict access to water to a small segment of the population (7). In this context, one pathway through which households may gain access to basic sanitation and hygiene infrastructure is through targeted intervention projects that construct facilities and provide technical or financial support for private or community use (8).

High-intensity public works are among the oldest and most widely recognized social-assistance programs designed to provide temporary employment and income to the poorest members of a community during periods of hardship (9, 10). The underlying principle of public-works programs is to transfer resources to poor households in exchange for labor, typically allocating the largest share of resources to wages while also supporting materials for infrastructure building and administrative costs through program design guidance (10). At present, public-works programs are one of the least-funded assistance programs in many African countries, including Somalia, South Sudan, Eritrea, Central African Republic, Burundi, Madagascar, Malawi, and Niger, where non-contributory benefits such as cash and food transfers receive greater priority (11, 12). However, these distributional schemes are limited to providing in-kind benefits. Public works, in contrast, can assist vulnerable households by providing benefits (cash or goods) in exchange for work while constructing or rebuilding facilities for the community (13, 14).

Madagascar represents a structurally relevant case for assessing the systemic interactions between infrastructure provision and public health dynamics across the Global South (12). It is one of the poorest countries worldwide, with a persistently high poverty rate of 80.7% in 2024. More than 39% of the children under 5 years of age in Madagascar suffer from stunting, and only 12.3% of the population has access to basic sanitation (1). Nearly 95% of the households use surface water and 85% of those use unprotected wells nationally (1). In the same year, the country was ranked 17th out of 193 countries in terms of exposure to natural disasters, owing to its geographical location and topography. This vulnerability, coupled with high variability in the socio-political and economic conditions in Madagascar, makes some zones more vulnerable than others. This vulnerability is even more extreme in the South (15), where the existing complementary measures to support the poorest after extreme climatic events or climate shocks are unevenly reaching the vulnerable poor (16). Therefore, the use of WASH facilities can be challenging during these extreme events. The number of children with malnutrition can also increase (17), since this number is directly linked to economic and nutritional poverty and indirectly linked to EED and poor living conditions (2, 5).

Since 1992, public-works programs, commonly known as *Haute Intensité de Main d'oeuvres* or high-intensity manual labor (HIMO), have been widely implemented across the country's regions as a strategic response to recurrent economic and climate crises (13, 18, 19). While these workfare initiatives primarily aim to provide low-wage employment to the most deprived households, they also facilitate the construction of essential community infrastructure, including water and sanitation facilities. Despite growing evidence from African and Asian contexts, the influence of these programs on household WASH access and child nutritional outcomes has not been sufficiently examined in Madagascar (20–22). Furthermore, while WASH practices may mediate the relationship between public works and child nutrition, indicating a complex intra-household dynamics, this mediating process remains understudied. Most impact evaluations have relied on direct path analysis, overlooking the indirect mechanisms through which workfare benefits translate into improved nutritional status (23).

Understanding the influence of this welfare dynamism on children is an important step to support the implications of public works for public health. Children represent the most vulnerable group in terms of health condition, empowerment, and intrinsic characteristics but are often omitted from the household benefits share (24). Child marginalization can be very contextual and may be exacerbated by intra-household inequality and cultural norms that favor elder household members in resource distribution within the family (25). Exploring household dynamics and the potential relationships among public works, WASH services, and child nutrition is essential, particularly in Southern Madagascar. Such insights are critical to informing policies aimed at reducing sociocultural inequality, alleviating economic poverty, and improving nutritional outcomes within regional programs.

**Figure 1:**
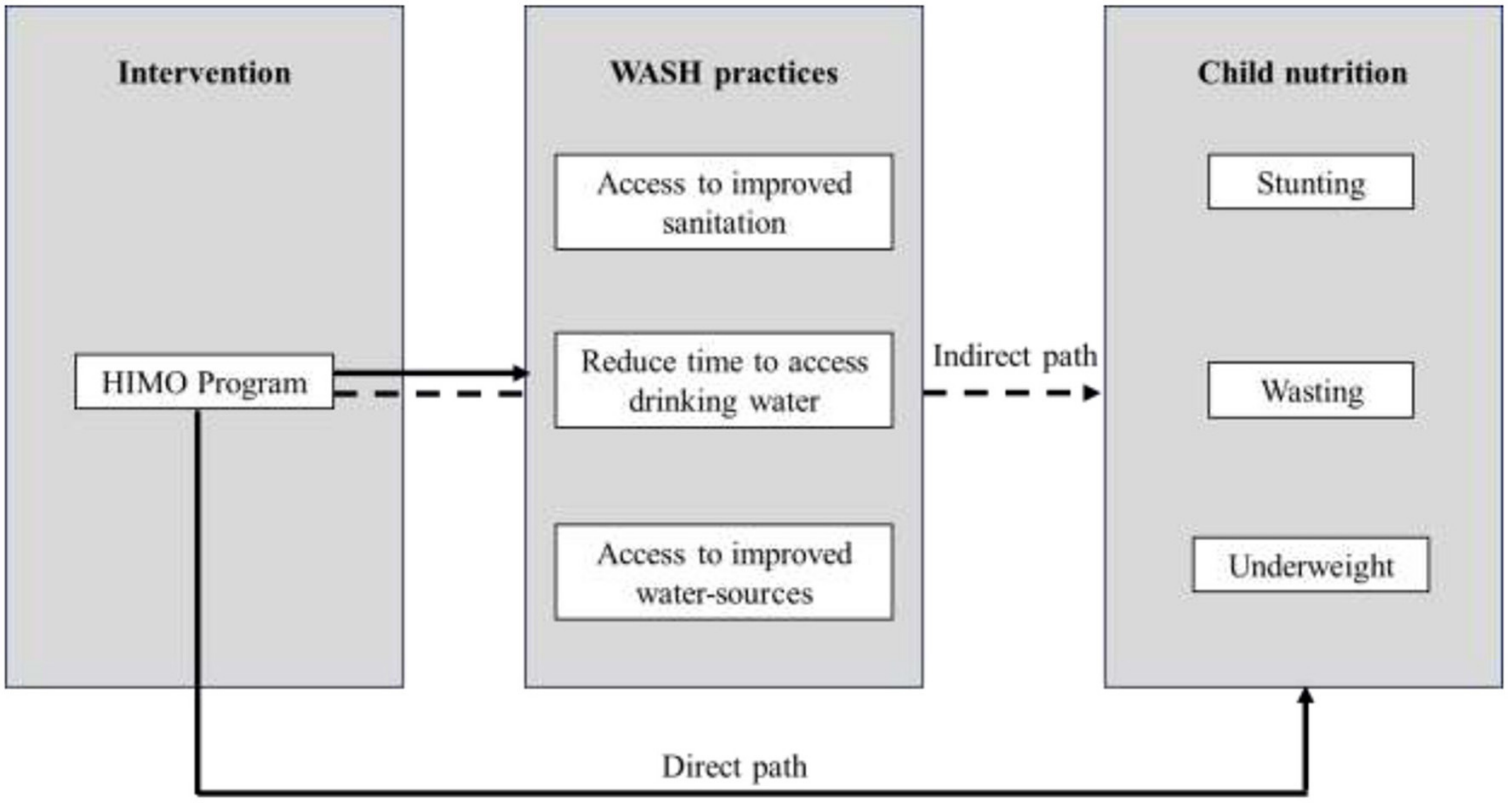
A pathway from workfare participation to improved child nutrition through mediation of WASH practices (Source: Authors).

This study evaluated the influence of public works on household access to improved water and sanitation and reduction of the travel time to drinking-water sources in Southern Madagascar. Furthermore, it elucidated the nutritional benefits for children and the mechanisms through which WASH services mediate these potential outcomes. While indirect factors, such as reduced disease incidence or increased time for childcare, likely contribute to the pathway between public works and nutrition, these were not explicitly modeled in the current theory of change (Figure 1), which focuses on WASH-related pathways. The evidence from this study can characterize the possible effects of household-targeted programs on children and increase the policy values of public works in the agenda.

This paper is organized as follows. Section 2 presents the data; the variables used in the analysis; and the econometric model employed for impact assessments. Section 3 illustrates the main results, including the treatment effects at the household and individual levels. Section 4 discusses this study's results, limitations, and policy implications. Finally, Section 5 provides the conclusions.

## 2 Materials and methods

### 2.1 Dataset

We used the Multiple Indicators Cluster Survey (MICS) datasets from United Nations Children's Fund and the National Institute of Statistics in Madagascar (INSTAT) to evaluate the effects of workfare program participation on household access to WASH facilities and children's nutritional outcomes. MICS is an open-source dataset that covers a wide range of topics, including sociodemographic characteristics, maternal and child health, education, WASH, and reproductive health across more than 120 countries (26). The 2018 Madagascar MICS-6 survey provides nationally representative data for households, children under 5 years of age, women, men, and adolescents. Due to the absence of more recent nationally comparable datasets, this study relied on the MICS-6 wave for its empirical analysis. Moreover, the design and delivery of public-works programs in Madagascar have remained largely stable since their scale-up in 2008, indicating that the structural conditions captured in 2018 continue to reflect a policy environment relevant to 2026 (12, 13).

This study uses the household, women, and under-five child datasets, merged to create a child-level analytical sample. The initial 13,355 observations were reduced to 5,879 after data cleaning and removing incomplete cases. To account for variations in household size, we created a composite weight by multiplying the household size by the child sampling weight, and applied this weight in all analyses. Our analysis focused on the southern half of Madagascar, covering nine regions across the provinces of Toliara, Fianarantsoa, and Mahajanga South, where socioeconomic precarity is higher than in the North. In this study, “Southern Madagascar” refers to this broader southern zone, not only to the Southwestern or Grand South areas used in other classifications (27).

To strengthen the analysis of HIMO's indirect effects on child nutrition, we incorporated geospatial covariates from the 2021 Madagascar Demographic and Health Surveys (DHS with GPS Data) and merged them with the MICS spatial dataset using the Quantum Geographic Information System software (QGIS). These covariates complemented existing MICS variables and helped address potential biases related to program assignment, confounding factors, and unobserved characteristics. The use of geospatial covariates, which has become increasingly common in recent studies (28), served here as complementary data to reduce selection bias and confounding in the Madagascar context.

### 2.2 Variable definitions and summary

#### 2.2.1 Outcome variables

We used the WHO guidelines for improved sanitation, water, and hygiene practices to construct the WASH variables (29). Improved sanitation was defined by the availability of toilet facilities with flush-to-piped sewer systems, flush-to-septic tanks, or flush-to-pit latrines with ventilated latrines, with or without slabs of open pits and composting toilets. In contrast, unimproved sanitation corresponded to facilities that flushed into open drains or unknown places, buckets, hanging toilets, hanging latrines, and open defecation. Improved water sources encompassed household water connections, public standpipes, boreholes, protected dug wells, protected springs, or rainwater collection (29). Unimproved water sources included unprotected wells, unprotected springs, surface water, vendor- and tanker truck-provided water (29). We converted the allocated time for accessing drinking water (in minutes) into a binary variable. In accordance with the WHO guideline for constructing WASH variables (29), the more time the household needs to get water (up to 30 min), the less likely it is to use improved water sources (29).

For children's nutritional outcomes, we used the WHO growth indicators for children under 5 years of age to assess the effect of project participation on the children's nutritional status (30). These include height-for-age *Z*-scores (HAZ) associated with stunting, weight-for-height *Z*-scores (WHZ) related to wasting, and weight-for-age *Z*-scores (WAZ), a reference for underweight. These scores measure the linear and weight growth of children relative to the expected values for their age.

Since our analysis focused on the influence of HIMO on child nutrition, we treated the child as the sampling unit rather than the household. In some cases, a household included more than one child under 5 years of age, and each child was surveyed separately in the child dataset. Consequently, children from the same household may have had different nutritional *Z*-scores while sharing identical household characteristics and exposure to HIMO. This clustering implied that observations were not fully independent, which we accounted for in the analysis.

#### 2.2.2 Treatment assignment and predictor variables

Information on participation in the HIMO program was drawn from the household dataset. Since MICS is not specifically designed to assess program participations or program effects on specific indicators, the targeting strategy used in the program is unknown. Public programs such as HIMO are often self- or community-targeted, which increases the risk of bias in selection. Omitted variable bias may also arise since unobserved factors such as economic, geographical, resilience to economic shocks, motivation, access to information, and social networks can influence household decisions and participation in the program.

On the basis of research conducted by Raholiarimanana and Ishida [16] concerning the targeting accuracy of social programs in Madagascar, we evaluated the main predictors of program participation using a probit model. Household socioeconomic and geographical variables such as household wealth index, sex of the household head, religious affiliation, possession of insecticide-treated nets (ITNs), and regional distribution were included in the model to predict HIMO participation (16). Since program assignments can occur at the village or commune level (13), we included regional indicators as fixed effects in our main equation to capture additional information related to geographical targeting and allow for inter-regional differences.

#### 2.2.3 Instrumental variables

To address bias arising from unobserved factors influencing household participation to the program, potential endogeneity in both the treatment and mediator equations, program placement correlated with pre-existing infrastructure or community characteristics, and the cross-sectional nature of the data, we employed an IV (28, 31). This strategy is widely endorsed in studies using cross-sectional datasets to evaluate mediation pathways (32, 33). Moreover, because our main outcomes relate to public health, concerns about endogeneity and the risk of bias are particularly salient.

Each instrument was selected on the basis of three criteria: relevance (a strong correlation with the endogenous variable), strict exogeneity (no direct effect on the outcome other than through the endogenous regressor), and contextual justification grounded in the institutional and geographic settings of Southern Madagascar. As with any IV-based analysis using cross-sectional data, exclusion restrictions could not be empirically tested and therefore relied on theoretical reasoning and the plausibility of the context.

##### 2.2.3.1 Instrument for program participation

Household contact with a social agent or community mobilizer during the 3 months preceding the survey was used as the instrument for HIMO participation. This variable follows an encouragement-design logic widely employed in development economics (34), whereby informational outreach increases program take-up without directly affecting outcomes. In rural Madagascar, where formal information channels are limited and literacy rates are low, social agents play a central role in disseminating information about program availability, eligibility, and enrollment procedures. Such contact substantially raises awareness of HIMO opportunities and lowers participation barriers, satisfying the relevance condition (first-stage *F*-statistic = 9.10).

Regarding exclusion, social agents involved in HIMO are primarily tasked with program mobilization and enrollment facilitation rather than direct provision of health education, sanitation services, or nutrition-related interventions. Unlike health extension workers or WASH promoters, these agents do not deliver behavior change communication, construct household facilities, or distribute health inputs. Thus, conditional on household wealth, regional fixed effects, and other socioeconomic controls, meeting with a social agent is unlikely to directly influence household WASH conditions or child nutritional status except through actual program participation. Although the first-stage *F*-statistic was close to, but slightly below, the conventional threshold of 10 suggested by Stock and Yogo, it remained near this benchmark and is supported by strong contextual justification and robustness checks (35).

##### 2.2.3.2 Instruments for WASH mediators

In the mediation analysis, we additionally addressed the potential endogeneity of WASH variables by using geospatial and housing-related instruments that captured the structural and environmental determinants of infrastructure access.

First, household housing status (homeownership and housing quality) was used as an instrument for access to improved sanitation. The secure tenure theory posits that households with formal ownership or durable housing structures have stronger incentives and capabilities to invest in permanent sanitation facilities than tenants or informal dwellers. Empirical evidence from sub-Saharan Africa has consistently shown that homeownership and housing quality are among the strongest predictors of improved sanitation. Studies using DHS data from Ethiopia showed that living in a modern or formal house more than doubled the odds of improved sanitation [adjusted odds ratio (OR) ≈ 2.09] in comparison with traditional housing (36). In Madagascar, Rahelinirina et al. (37) documented that informal settlements and rental housing exhibit substantially lower sanitation coverage. In our data, housing status revealed a strong first-stage relationship with sanitation access (*F*-statistic = 21.62). Conditional on household wealth, education, regional location, and program participation, housing tenure is unlikely to exert a direct biological or behavioral effect on child nutrition, supporting the exclusion restriction.

Second, we used the enhanced vegetation index (EVI) as an instrument for the time required to access drinking-water sources. The EVI captures vegetation density and greenness, which are closely linked to hydrological conditions, groundwater availability, and the spatial distribution of water points (38, 39). Areas with higher EVI tend to have more abundant and proximate water sources, whereas arid zones involve longer water-collection times. A comprehensive global analysis of drinking-water access in low- and middle-income countries identified biogeographic and environmental covariates, including vegetation-related indicators such as EVI, as major contributors to predicting the availability of drinking water on household premises (40). This relationship was strongly confirmed in our sample (*F*-statistic = 45.30). Although vegetation may also be correlated with agricultural productivity, we mitigated this concern by controlling for household wealth, regional fixed effects, and program participation, and by focusing on anthropometric *Z*-scores, which reflect cumulative nutritional status rather than short-term dietary fluctuations.

Third, we employed nighttime light intensity as an instrument for accessing improved drinking-water sources. Nighttime lights provide a satellite-based proxy for local infrastructure density, urbanization, and service provision, and this approach has been validated in multiple African contexts and extensively documented in the literature on economic development (41). In low-income settings, brighter areas consistently exhibit higher coverage of piped water, protected wells, and public standpipes (42, 43). The exceptionally strong first-stage relationship in our data (*F*-statistic = 183.5) confirmed the relevance of this instrument to the study. Although nighttime lights are also correlated with broader economic development, a concern widely discussed in the literature, our use of an explicit condition for household wealth and regional fixed effects isolated residual variation that primarily reflects the availability of spatial infrastructure rather than household-level socioeconomic status. Under this conditioning set, nightlight intensity is plausibly exogenous to child nutritional outcomes, except through its influence on water infrastructure.

Taken together, the convergence of strong first-stage relationships, well-established theoretical mechanisms, extensive socioeconomic controls, and contextual relevance to Southern Madagascar enhanced the credibility of our IV strategy. Although exclusion restrictions remain fundamentally untestable, the consistency of these elements, which was supported by robustness checks, strengthened the credibility of our IV strategy in identifying the associations between HIMO participation, WASH improvements, and child nutritional outcomes.

### 2.3 Estimation strategy

Our mediation analysis used the potential-outcomes framework to estimate how HIMO affects WASH and nutrition indicators (44, 45). This framework relies on a counterfactual idea: we estimated what the outcomes of treated children would have been if they had not received the program, and what the outcomes of untreated children would have been if they had received it. By comparing these predicted counterfactual outcomes, we could identify the direct effect of HIMO and the part of the effect that works through the mediators. For clarity, we present the main equations used for the direct and indirect effects in the paper, while the full technical details are provided in the Supplementary materials.

#### 2.3.1 Analysis of direct treatment effects

This analysis evaluated the total effect of the program on the outcomes of interest. The analysis of the treatment effects on the potential outcome variables is given by the following equation:


$$\begin{array}{l} Y_{ir}\;=\;\beta_{0}+\beta_{1}Treatment_{ir}+\;\beta_{2}X_{ir}+\gamma_{r}+\varepsilon_{ir} \end{array}$$ (1)

Where *Y*_*ir*_ represents the outcome of the individual *i* living in the region *r*, β_1_ is the coefficient of interest, *X*_*i*_ is a vector of control variables, γ_*r*_ is the region-fixed effects, and ε_*ir*_ relates to the error term.

In this expression, the treatment can take a value of 0 if the sample unit did not participate in the program, and 1 otherwise. In the presence of endogeneity, the treatment variable becomes correlated with the error term ε_*ir*_ and requires an IV approach to address the issue. For such cases, the treatment variable is specified as follows:


$$\begin{array}{l} Treatment_{ir}=\;{\alpha_{0}+\;\alpha_{1}IV_{ir}^{T}+\alpha }_{2}X_{ir}+\;\gamma_{r}+\nu_{ir} \end{array}$$ (2)

Where $IV_{ir}^{T}$ is the instrument for the treatment. $IV_{ir}^{T}\;$must not be related to any of the variables included in the treatment equation. Thus, Equations 1 and 2 form a system of equations that are estimated simultaneously to assess the endogenous treatment effects on WASH and nutritional outcomes:


$$\{\begin{array}{l} Treatment_{ir}=\;\alpha_{0}+\;\alpha_{1}IV_{ir}^{T}+\alpha_{2}X_{ir}+\;\gamma_{r}+\nu_{ir}\\ Y_{ir}\;=\;\beta_{0}+\beta_{1}{\hat{Treatment}}_{ir}+\;\beta_{2}X_{ir}+\gamma_{r}+\varepsilon_{ir} \end{array}$$ (3)

Where ${\hat{Treatment}}_{ir}$ is the predicted value of treatment from the first stage and which is uncorrelated to ε_*ir*_.

The resulting marginal effect from Equation 3 corresponds to the local average treatment effect (LATE). The LATE represents the treatment effect for compliers, i.e., individuals who received the treatment because they were influenced by the IV. When the treatment is exogenous, Equation 1 alone is sufficient to estimate the effects of the program on the outcomes, and the marginal effect corresponds to the average treatment effect (ATE) rather than LATE.

#### 2.3.2 Mediation and indirect-effect analysis

If any of the household-level outcome variables (WASH practices in the present study) is significantly affected by the program, it becomes a potential mediator and is tested in a mediation framework. The Equation 4 for children's nutritional outcomes is then specified as follows:


$$\begin{array}{l} Y_{ir}\;=\;\beta_{0}+\beta_{1}Treatment_{i}+\;\beta_{2}X_{ir}+\beta_{2}Mediator_{ir}+\gamma_{r}+\varepsilon_{ir} \end{array}$$ (4)

If the treatment in endogenous, Equation 4 becomes Equation 5 and is expressed as follows:


$$\{\begin{array}{l} Treatment_{ir}=\;\alpha_{0}+\;\alpha_{1}IV_{ir}^{T}+\alpha_{2}X_{ir}+\;\gamma_{r}+\nu_{ir}\\ Y_{ir}\;\;=\;\beta_{0}+\beta_{1}{\hat{Treatment}}_{i}+\;\beta_{2}X_{ir}+\beta_{2}Mediator_{ir}+\gamma_{r}+\varepsilon_{ir} \end{array}$$ (5)

Some WASH-related variables may also be endogenous to children's *Z*-scores and require IV techniques to correct for endogeneity (Equation 6). In such cases, the WASH equation is specified as follows:


$$\begin{array}{l} Mediator_{ir}=\;\delta_{0}+\;\delta_{1}IV_{ir}^{M}+\delta_{2}X_{ir}+\;\gamma_{r}+\eta_{ir} \end{array}$$ (6)

When both the treatment and the mediator are endogenous, the system of equations for mediation analysis is defined in Equation 7 as:


$$\{\begin{array}{l} Treatment_{ir}=\;\alpha_{0}+\;\alpha_{1}IV_{ir}^{T}+\alpha_{2}X_{ir}+\;\gamma_{r}+\nu_{ir}\\ Mediator_{ir}=\;\delta_{0}+\;\delta_{1}IV_{ir}^{M}+\delta_{2}X_{ir}+\;\gamma_{r}+\eta_{ir}\\ Y_{ir}\;=\beta_{0}+\beta_{1}{\hat{Treatment}}_{ir}+\;\beta_{2}X_{ir}+\beta_{2}{\hat{Mediator}}_{ir}+\gamma_{r}+\varepsilon_{ir}\; \end{array}$$ (7)

Where ${\hat{Treatment}}_{ir}$ and ${\hat{Mediator}}_{ir}$ are predicted values obtained from the first stage and are uncorrelated with the error term ε_*ir*_.

We analyzed the data using StataNow v.19, a software developed by StataCorp LLC, USA, applying the extended regression commands *eregress* for continuous outcomes and *eprobit* for binary outcomes.

#### 2.3.3 Effect calculations

**Figure 2:**
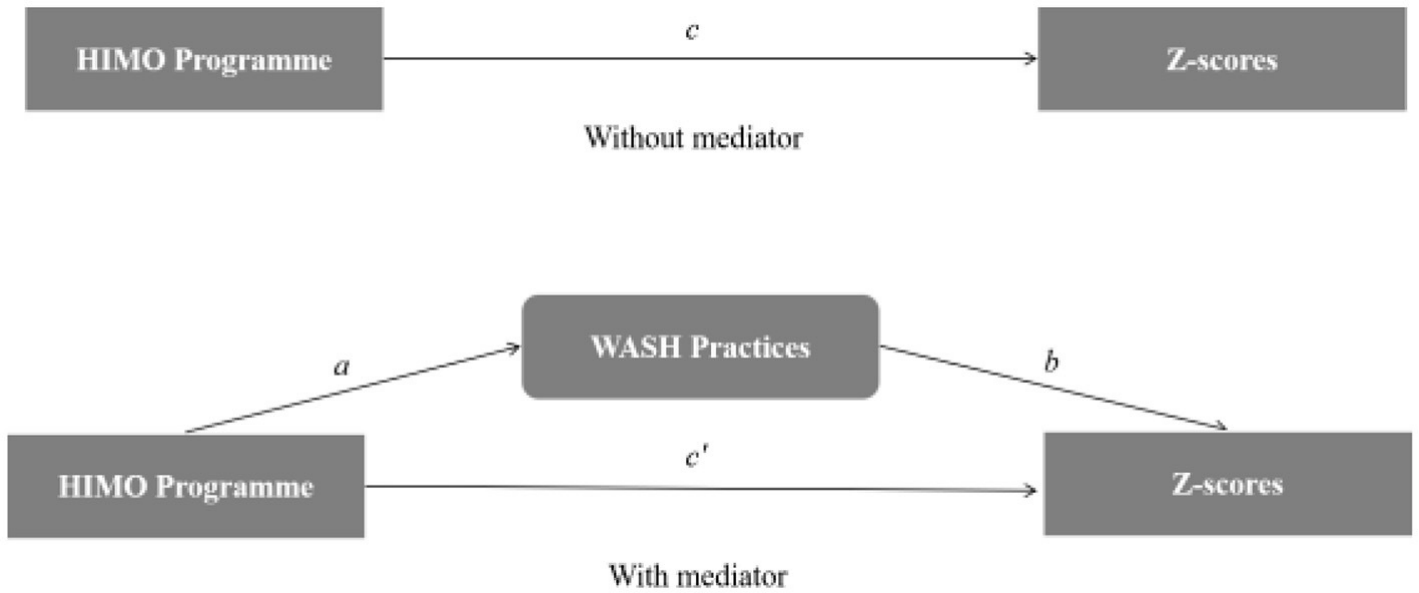
Estimation strategy of direct and mediated effects of HIMO on child nutrition.

Figure 2 summarizes the calculation strategy for estimating total, direct, mediated, and indirect effects. The variable *c* denotes the total direct effect from the direct treatment effect approach, derived from Equations 1 or 3. The coefficient *c*′ represents the direct effect of the program after accounting for the mediator, obtained from Equations 5 or 7. The coefficient *a* denotes the effect of the treatment on the mediator, and *b* represents the effect of the mediator on the outcome. The mediated (indirect) effect is the product *ab*, and the proportion mediated is calculated as *ab*/(*ab*+*c*′*)*. The indirect effect can also be computed as the difference between the total and direct effects, i.e., *c* and *c*′.

## 3 Results

The HIMO program benefited 19.42% of the children under 5 years of age in Southern Madagascar. Among these, 15.72% belonged to households with access to improved sanitation, and 26.24% belonged to households with access to improved drinking-water sources. More than 60% of the children, however, belonged to households with access to the nearest drinking-water source within a 30-min walk. Children showed normal growth in height and weight metrics. The mean *Z*-scores for stunting, underweight, and wasting were all situated above the −2 threshold, indicating normal stature of children in Southern Madagascar (cf. Table 1). However, the prevalences of stunting (39.52%) and underweight (25.83%) were relatively high, and a moderate prevalence of wasting (7.25%) was observed in the area.

**Table 1:** Definitions and summary of variables.

Category	Variable	Measurement and definition	Scale	Mean (SD) or % (*N*)
Treatment variable	HIMO participation	Exposition of 0- to 59-month-old children to the program (binary)	0 and 1	19.42 (1142)
Outcome variables	Improved sanitation	Access of the household to toilet facilities with flush-to-piped sewer systems, flush-to-septic tanks, or flush-to-pit latrines with ventilated latrines, with or without slabs of open pits and composting toilets (binary)	0 and 1	15.72 (924)
	Duration of access to drinking- water sources	Allocated time for accessing drinking water, in up to 30 min or less (binary)	0 and 1	60.41 (3551)
	Improved drinking-water sources	Household access to improved drinking-water sources such as household connections, public standpipes, boreholes, protected dug wells, protected springs, and rainwater collection (binary)	0 and 1	26.24 (1543)
	HAZ	Children's height-for-age *Z*-score (HAZ) associated with stunting (continuous)	−6 to 5.72	−1.597 (1.38)
	WAZ	Children's weight-for-age *Z*-score (WAZ) associated with underweight (continuous)	−6 to 6	−1.295 (1.10)
	WHZ	Children's weight-for-height *Z*-score (WHZ) associated with wasting (continuous)	−6 to 6	−0.536 (1.03)
Control variables	Wealth index	Composite-based poverty index included in the MICS dataset, categorized into five quantiles: from the poorest to the richest (from 1 to 5)	1st quantile	41.86 (2461)
			2nd quantile	28.19 (1657)
			3rd quantile	17.11 (1006)
			4th quantile	7.57 (445)
			5th quantile	5.27 (310)
	Regional distribution	Regional allocation based on rural Southern Madagascar (categorical)	Haute Matsiatra	11.15 (655)
			Amoron'i Mania	7.79 (310)
			Vatovavy Fitovinany	12.87 (757)
			Ihorombe	3.28 (193)
			Atsimo Atsinanana	13.25 (779)
			Atsimo Andrefana	19.76 (1162)
			Androy	14.83 (872)
			Anosy	9.68 (569)
			Menabe	7.38 (434)
	Religion	Faith-based affiliation of the household (categorical)	No religion	39.01 (2293)
			Christianity	46.63 (2741)
			Other religion	14.36 (844)
	Sex of household head	Sex of the household head, referred by men (binary)	0 and 1	83.98 (4937)
	ITN possession	Possession of insecticide-treated bed nets (binary)	0 and 1	94.49 (5555)
Instrumental variables	Meeting with a social agent	Meeting with a social mobilizer or facilitator during the last 3 months (binary)	0 and 1	23.45 (1378)
	Housing status	Housing status of the household (ref: tenant vs. homeowner, binary)	0 and 1	90.43 (5316)
	Enhanced vegetation index	Average vegetation index value in a year, ranging from least vegetation to most vegetation (continuous)	0.167–0.506	0.290 (0.08)
	Nightlight composite	Geospatial covariate describing the nighttime luminosity in the zone in composite cloud-free radiance values (continuous)	0–2.236	0.061 (0.31)
Observation	0–59 months children	Data merged from MICS' household, women, and children dataset and DHS spatial covariates		5879

All values presented in Table 1 are weighted and scaled using a composite weight, calculated as the child weight provided in the MICS dataset multiplied by the household size, and incorporating the PSU and strata specifications defined in the STATA's svyset command for survey estimates.

The HIMO program is likely to target all wealth categories except the richest group (cf. Table 2). The sex and religious affiliation of the household head also significantly affected the program selection. No difference in program coverage and geographical selection was observed across the Southern regions, except for the Vatovavy Fitovinany region. Households who met with a social agent within the last 3 months had higher likelihood of participating in the program than those who did not. Program selection and the main characteristics of households reached by HIMO are presented in Table 2.

**Table 2:** Probit model for treatment selection.

Predictor variables	Without instrumental variable	With instrumental variable
Religion (ref: no religion)		
Christianity	0.251^*^ (0.10)	0.222^*^ (0.10)
Other religions	0.199 (0.12)	0.155 (0.12)
Economic status (ref: richest)		
Poorest	0.901^***^ (0.20)	0.895^***^ (0.19)
Lower-middle poor	0.912^***^ (0.19)	0.900^***^ (0.19)
Middle poor	0.872^***^ (0.20)	0.840^***^ (0.20)
Upper-rich	0.952^***^ (0.23)	0.954^***^ (0.23)
Regions (ref: Haute Matsiatra)		
Amoron'I Mania	−0.360 (0.24)	−0.369 (0.24)
Vatovavy Fitovinany	0.020 (0.24)	0.073 (0.24)
Ihorombe	−0.631^*^ (0.27)	−0.631^*^ (0.27)
Atsimo Atsinanana	−0.189 (0.25)	−0.186 (0.25)
Atsimo Andrefana	0.194 (0.27)	0.201 (0.27)
Androy	0.180 (0.24)	0.160 (0.24)
Anosy	−0.093 (0.28)	−0.253 (0.28)
Menabe	−0.484 (0.29)	−0.501 (0.29)
Head of household (ref: male)		
Female	−0.216^*^ (0.09)	−0.207^*^ (0.09)
Insecticide-treated net possession (ref: no)		
Yes	0.270 (0.19)	0.248 (0.19)
Meeting a social agent (ref: no)		
Yes		0.305^***^ (0.08)
Constant	−1.178^***^ (0.26)	−2.078^***^ (0.31)
Observations	5879	5879

^***^p < 0.001, ^**^p < 0.01, ^*^p < 0.05; standard errors are in parentheses. ref, reference. Meeting with a social agent is the instrumental variable for the treatment.

The access of children aged under 5 years (U5) to improved sanitation facilities significantly increased by 55.1% if the program was distributed universally. An improvement of 15.7% was observed among families with children who directly participated in the program. At the end of the program, more than 67.7% of these families were likely to have access to improved sanitation. The time allocated to access drinking-water sources also decreased significantly. Under universal distribution, the prevalence of households with U5 children having access to water sources within less than 30 min would increase by 42.3%, while it would increase by 56.8% for targeted programs. After program exposure, more than 91.6% of U5 children had access to drinking-water sources within a 30-min walk. However, the program showed no significant effect on the access of children to improved water sources. Nevertheless, nearly 25%−34% of the Southern households had access to improved drinking-water sources, with higher estimates observed among the treatment group (cf. Table 3).

**Table 3:** Total direct effects of the public-works programs on household WASH practices.

WASH practices	(L)ATE	ATET (a)	Potential outcome means	
			Untreated	Treated
Improved sanitation	0.551^***^ (0.13)	0.157^***^ (0.03)	0.126^***^ (0.01)	0.677^***^ (0.13)
Time to access drinking water	0.423^***^ (0.02)	0.568^***^ (0.04)	0.493^***^ (0.02)	0.916^***^ (0.01)
Improved water sources	0.091 (0.16)	0.064 (0.12)	0.250^***^ (0.03)	0.341^***^ (0.14)

^***^p < 0.001, ^**^p < 0.01, ^*^p < 0.05; standard errors are in parentheses. (a) is as referred in Figure 2 and corresponds to the effect of the treatment on the mediator. Local average treatment effect (LATE) values are provided for the improved sanitation and time to access drinking-water variables as the treatment was found to be endogenous. Average treatment effect (ATE) values are provided for improved water sources as the treatment was found to be exogenous. These estimates are drawn from counterfactual estimates under the potential outcome framework, and are obtained after controlling for HIMO predictors and an instrument variable in case of treatment endogeneity.

The total direct effects corresponding to the ATE or LATE and average treatment effect on the treated (ATET) are presented in Table 3. Information on adjusted coefficients in each equation stage can be found in Supplementary Table SI, alongside the intercorrelations between each stage's error terms.

Growth improvement was observed among children after HIMO program exposure. Children's HAZ, WAZ, and WHZ scores significantly increased by 1.36, 0.82, and 0.86 unit points, respectively. Notable improvement was also observed in the treated group, with the potential outcome scores largely surpassing those of the untreated group (cf. Table 4). Adjusted coefficients and intercorrelations in error terms between treatment and children's nutritional outcome variables can be found in Supplementary Table SII.

**Table 4:** Total direct effects of public-works programs on children's nutritional outcomes.

Nutrition outcomes	LATE	ATET (c)	Potential outcome means	
			Untreated	Treated
HAZ (stunting)	1.365^***^ (0.28)	1.329^***^ (0.27)	−1.855^***^ (0.08)	−0.489^**^ (0.22)
WAZ (underweight)	0.820^***^ (0.27)	0.787^***^ (0.26)	−1.447^***^ (0.07)	−0.627^**^ (0.22)
WHZ (wasting)	0.857^***^ (0.23)	0.810^***^ (0.22)	−0.699^***^ (0.05)	0.164 (0.19)

^***^p < 0.001, ^**^p < 0.01, ^*^p < 0.05. Standard errors are in parentheses. (c) as referred to in Figure 2 corresponds to the total direct effect when calculating mediation effects. Local average treatment effect (LATE) values are provided for HAZ, WAZ, and WHZ since the treatment was found to be endogenous. These estimates are drawn from counterfactual estimates under the potential outcome framework, and are obtained after controlling for HIMO predictors and treatment-related instrumental variable.

In Table 5, the direct effects of the treatment on children's *Z*-scores, under potential mediator influences, were significant at the 0.1% level. Improved sanitation mediated the treatment effects on HAZ and WAZ by 1.287 and 0.708, respectively. However, no mediating effects of improved sanitation on WHZ were observed. Instead, the time to access drinking-water sources was more likely to influence WHZ with a significant contribution of 0.988. Nonetheless, this mediator variable did not show any significant effects on children's HAZ and WAZ. Adjusted coefficients and error-term correlations between the outcome, the treatment, and the mediator equations can be found in Supplementary Table SIII for improved sanitation and in Supplementary Table SIV for time to access drinking-water.

**Table 5:** Direct and marginal effects of public-works programs on child nutritional status through WASH.

Mediator effects	HAZ	WAZ	WHZ
Improved sanitation			
Marginal effect (b)	1.287^***^ (0.21)	0.708^**^ (0.22)	−0.055 (0.05)
Direct effect (c′)	1.222^**^ (0.43)	0.759^**^ (0.35)	0.824^***^ (0.21)
Time to access drinking-water sources			
Marginal effect (b)	−0.026 (0.05)	−0.036 (0.04)	0.988^***^ (0.20)
Direct effect (c′)	1.328^***^ (0.27)	0.784^**^ (0.26)	0.992^***^ (0.26)

^***^p < 0.001, ^**^p < 0.01, ^*^p < 0.05; standard errors are in parentheses. (b) represents the marginal effects of WASH-related variables on children's Z-scores, while (c′) corresponds to the program's direct effect after controlling for the mediator, treatment predictors, treatment-related instrument variable, and mediator-related instrument variable in a case of mediator endogeneity.

The results from the mediation effects of WASH-related variables under the public-works program are presented in Table 6. The effect of using sanitation facilities corresponded to 14% of the total program effect on the stunting indicator, and 12% on the underweight indicator. Time to access drinking water contributed to nearly 36% of the total direct effect of HIMO on wasting *Z*-scores. Although both the average causal mediation effects (ab) and the difference between coefficients (c-c′) represent indirect treatment effects, they were not similar across the *Z*-scores. Such a difference was likely driven by the non-linear nature of the models, the addition of the covariates, and the correction for treatment and mediator endogeneities. The mediated effects reported in this study are related to the product of coefficients (ab) while the difference (c-c′) is displayed only for information.

**Table 6:** Mediation effects of WASH-related variables on children's nutritional indicators.

Mediators	Direct and mediated effects	HAZ	WAZ	WHZ
Improved sanitation	Average causal mediation effects (ab)	0.199	0.111	0
	Mediated effects in % [ab/(ab+c′)]	14.00%	12.77%	0%
	Mediated effects in % (ab/c)	14.97%	14.12%	0%
	Difference between coefficients (c-c′)	0.107	0.028	−0.014
	Total direct effects (c)	1.329	0.787	0.81
Time to access drinking-water sources	Average causal mediation effects (ab)	0	0	0.561
	Mediated effects in % [ab/(ab+c′)]	0%	0%	36.13%
	Mediated effects in % (ab/c)	0%	0%	69.28%
	Difference between coefficients (c-c′)	0.001	0.003	−0.182
	Total direct effects (c)	1.329	0.787	0.810

All estimates are significant at the 0.1% level. Reported mediated (indirect) effects in the main text are represented by the average causal mediation effects (ab).

**Figure 3:**
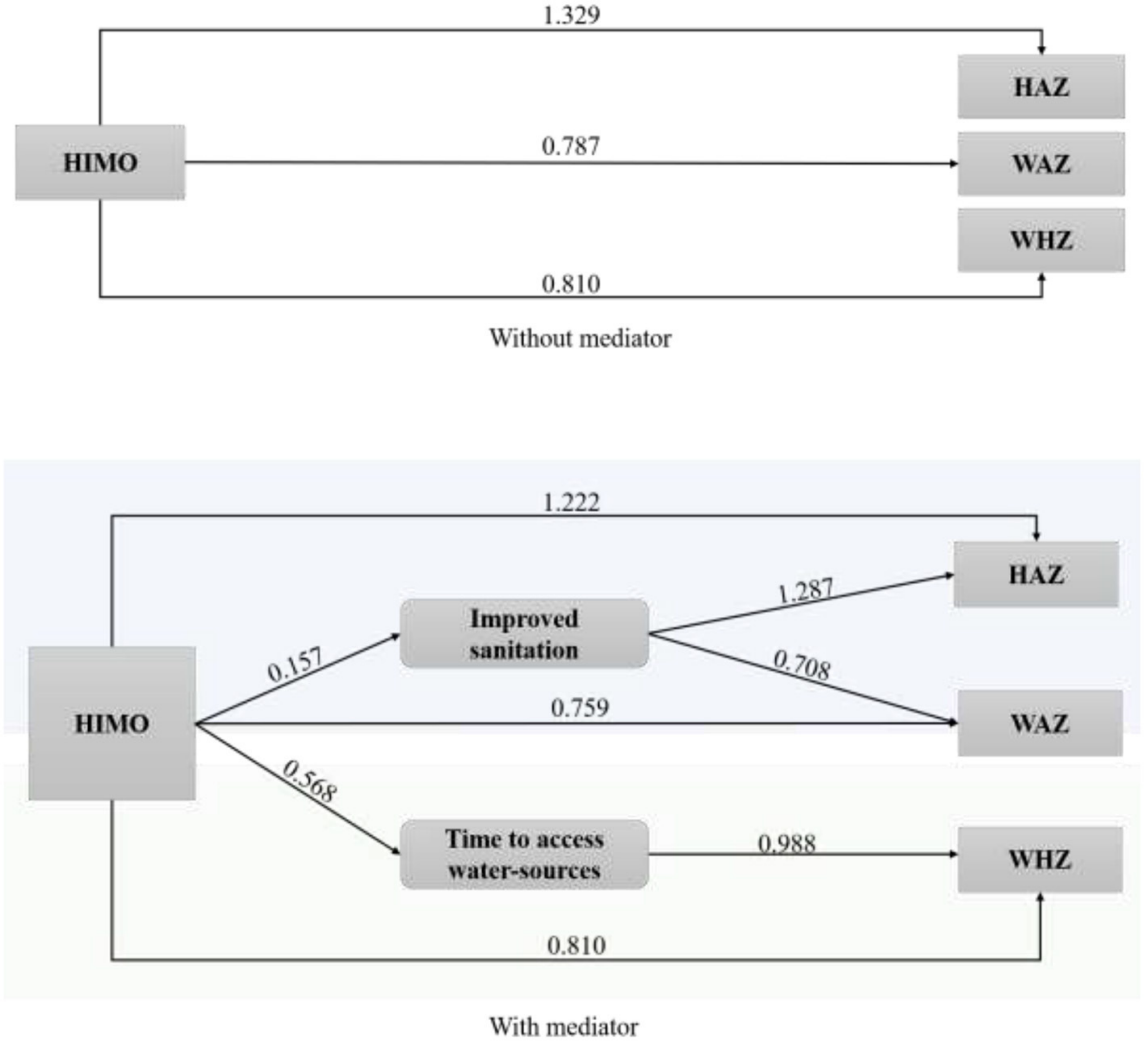
Direct and mediated effects of HIMO on child nutritional outcomes, comparing models without and with sanitation and water-access mediators.

Figure 3 illustrates the effectiveness of the public-works program in improving children's nutritional outcomes in Southern Madagascar. It provides evidence showing that improved sanitation significantly mediates the positive effects on stunting (HAZ) and underweight (WAZ), while the time required to access drinking-water sources mediates the effect on wasting (WHZ).

## 4 Discussion

This study examined the associations between public-works program participation and child nutrition in Southern Madagascar, focusing on the potential mediating role of WASH indicators. Under these programs, households with children were more likely to gain access to improved sanitation and spend less time reaching the nearest drinking-water source. These improvements accounted for 14% of the total effect of the program on stunting *Z*-scores, 12% of the effect on underweight *Z*-scores, and 36% of the effect on wasting *Z*-scores. Given the high prevalence of child malnutrition in the area, public-works programs offer an alternative for households to cope with recurrent socioeconomic shocks, access improved WASH facilities, and support better nutritional and health statuses for their children. These findings offer useful policy insights for improving public health indicators such as WASH access and children's *Z*-scores under social protection schemes in fragile contexts. Unlike many previous studies, this analysis explicitly accounted for endogeneity in both the treatment and the mediator.

### 4.1 Direct effects of HIMO participation on WASH conditions

Our direct-effect analysis indicated that public-works programs likely reduce barriers to basic sanitation and water infrastructure in Southern Madagascar. Amid disasters such as flooding, labor-intensive perspectives programs use the local workforce and the local material supply to sustain the livelihood of the population and help optimize the households' WASH conditions (13, 14, 46). It is during disaster events that the risk of environmental borne diseases becomes imminent, making the prevention of disease spread and the management of good hygienic conditions challenging. Developing countries, including Madagascar, often lack the basic infrastructure that is supposed to sustain them, and public works programs present one alternative to support WASH facilities and enhance public health. From public works to improved WASH conditions, there are several pathways to consider.

First, access to improved sanitation facilities may already fall within the mandate of the public works agendas (47). In Madagascar, decisions regarding the construction of sanitation infrastructure are typically made at the commune level, as illustrated by the national HIMO program implemented across all 23 regions (13, 48, 49). Municipal administrations assess local needs, submit requests to the national social protection administration, and receive subsidies accordingly. Public works budgets, drawn from both regular and emergency funds, enable local administrative units to implement specific projects within their jurisdictions (13, 49). Other initiatives operate under special development programs supported by non-governmental donors or agencies, targeting domains such as WASH-related behaviors, women's empowerment, primary education, or environmental promotion. Some programs, such as ACTP-Asa Avotra Mirindra, have integrated training sessions into their agendas, covering themes ranging from technical assistance to child nutrition (14). This program encouraged mothers to attend nutrition community sites or health facilities in addition to participating in public works activities (14). Infrastructure developed through these projects is generally shared and accessed collectively by the community. But most importantly, the management of newly built infrastructure by local communities is often overlooked after implementation. These facilities require periodic servicing, maintenance, and cleaning (47). Such management is difficult to ensure in remote settings (50), particularly during periods of crisis or natural disasters. Greater attention to the maintenance and proper use of these infrastructures is therefore essential to guarantee their continued availability and accessibility.

Second, household access to improved WASH facilities can be considered a “by-product” of program exposure, especially when the program has the potential to improve household livelihoods directly, ease food insecurity in the short run, and allow households to interact with social agents. Households that have participated in social interventions have been reported to develop greater awareness of their wellbeing and are often more eager to maintain it by adopting positive behaviors (51). Beneficiary households may therefore develop a sense of necessity regarding the access and use of toilets and may decide to use readily available sanitation facilities or to build their own. Nelson et al. (52) and Malolo et al. (53) reported similar results in their studies, supporting the impacts of social programs on household decision and behaviors related to WASH infrastructure. Other studies in other contexts confirm that beneficiary households get better-off after program participation and decide to invest in infrastructure such as sanitations to materialize the profits (54–57). Improvements in WASH conditions and practices can therefore be attributed to the overall enhancement of household wellbeing under the program.

Third, a closer look toward a targeting and inequality lens suggests that the observed improvements in WASH access may partly stem from earlier program mistargeting. Evidence from the HIMO program in Southern Madagascar highlights substantial mistargeting: with the exception of the wealthiest group, nearly all households were likely to receive program benefits (16). Miller-Petrie et al. (58) stipulated the burden of poverty as the main socioecological drivers of WASH choices of households in Northwest Ecuador. In this context, the gains in access to improved facilities may reflect underlying wealth gradients rather than program effects *per se*, as better-off households in Madagascar are more likely to own private latrines (59). Regardless of the funding channel, improvements in WASH conditions align with the principles of highly labor-intensive approaches and humanitarian interventions to support vulnerable households financially and materially during critical periods by relying on local labor and local material supply (60, 61). The construction of basic community infrastructure also contributes positively to local development and provides income-earning opportunities for low-skilled workers and low-income households.

### 4.2 Direct effects of HIMO participation on child nutritional status

Public-works programs likely help prevent, protect, and reduce the prevalence of malnutrition in the area, as stipulated by the direct analysis estimations of the *Z*-scores of children. Improvements in children's growth indicators were observed both at the population and treated household levels, demonstrating the public health relevance of the HIMO program. The HAZ score, which reflects long-term physical development during the first 1,000 days and beyond, is a key indicator of cumulative growth conditions (62). Children with low HAZ scores have shorter height compared to other children of the same age and may be affected by stunting, an invisible form of undernutrition. Stunting is a complex multidimensional phenomenon resulting from repeated exposure to poor diets and/or adverse health conditions, preventing children from achieving their full growth potential. It is irreversible and has several consequences for children's physical, emotional, and cognitive development (62). Conversely, the weight-related *Z*-scores such as WAZ (for underweight) and WHZ (for wasting), reflect children's body mass development and are strong indicators of current food deprivation, particularly among children suffering from acute malnutrition (62). Wasting results from severe food deprivation, such as during famine, is often compounded by disease. Unlike stunting, which is irreversible, wasting can lead rapidly to death and requires immediate medical interventions (38). It is characterized by low body mass and may be accompanied by edema and other physical dysfunctions. Underweight reflects the immediate nutritional status of children and results from a moderate to severe food deprivation occurring within a short period. It is manifested by low weight-for-age, and can be addressed by providing children with adequate and nutritious food.

The direct effects of public works on child nutrition can be twofold: the short-run effect arising from positive dynamics in the benefit share, meaning a direct consumption of resources by children, and a long-run effect resulting from the compounding benefits reflected in household livelihoods, socioeconomic conditions, and the broader environment, ultimately benefiting children. In contrast to individual-targeted programs that benefit only the principal recipients, household-targeted social programs such as the HIMO program are likely to have a wider impact and can strengthen household adaptive capacity to shocks while enhancing food and nutrition security. The short-run perspective suggests that the benefits generated by these programs reach other household members, particularly children, indicating dynamism in the intra-household allocation of resources. In Southern Madagascar, where children are often disadvantaged due to cultural norms that prioritize older adult members and men (63–65), they may benefit directly from changes in household economic conditions or indirectly through improved living standards and better access to basic services. The impact on wasting is particularly noteworthy, given that both wasting prevalence and the implementation schedule of public works programs share a temporal dimension that links them closely. The predictability of public works during the lean season or in the aftermath of shocks therefore helps prevent acute nutritional deterioration among children.

The long-run perspective stipulates that public works can support sustained development by enabling households to access productive inputs, materials, and livelihood assets during lean seasons or post-disaster periods (18). Global evidence demonstrates that such programs can help households overcome barriers to essential services including sanitation, piped water, informal financial services, village savings and loan associations, health care, and even improvements in vegetation and biophysical conditions (66–69). In that case, the public works initiatives traditionally viewed as short-term safety nets can become transformative and influence multiple outcomes within a single intervention, including child nutrition.

The positive effects of public works on stunting, wasting and underweight indicators observed in this study are consistent with previous studies. Evidence from Ethiopia suggests that the Productive Safety Net Program (PSNP) implemented between 2006 and 2009 improved the nutritional status of children in participating households by acting as a safety net and preventing nutritional vulnerability (21). Despite program's unconditional nature, various studies have shown that the PSNP's effects on child nutrition may stem from increases in household consumption and income (21), improved food security, and household investments in productive assets such as livestock (70). Evidence from Malawi also confirms the effectiveness of both combined and individual social protection interventions including cash, school feeding, supplementary feeding, food-based, and public works in improving household food security and reducing stunting among children (71). Zembe-Mkabile (72) highlighted that the main drivers of malnutrition in Sub-Saharan Africa extend beyond food and diets, encompassing household-level dynamics, maternal factors, the external environment, and social protection, and emphasizing their importance as key levers for improving maternal and child nutrition. Landin et al. (73), in their longitudinal analysis of 46 low- and middle-income countries, found that over two decades, social protection interventions significantly reduced the prevalence of stunting, wasting, and toddler mortality. Other authors have argued that the successful reduction of malnutrition requires multisectoral implementation involving districts, communes, and villages, as demonstrated in Indonesia (74), Peru (75), Senegal (76), and Ethiopia (77). However, the magnitudes of our estimated associations (HAZ +1.36, WAZ +0.82, and WHZ +0.86 SD) appear substantially larger than those reported for comparable public-works and cash-transfer programs in sub-Saharan Africa, where stunting reductions of 0.10–0.30 SD are more typical (21, 70). This difference may reflect the local average treatment effect (LATE) interpretation of our IV estimates (i.e., effects for compliers), contextual specificity in Southern Madagascar, and the relatively weak first stage of the treatment instrument (*F* = 9.10). These findings should therefore be interpreted as upper-bound suggestive estimates rather than directly comparable causal magnitudes.

### 4.3 Mediating role of WASH conditions in the relationship between HIMO participation and child nutritional status

The mediation analysis further refines this discussion by demonstrating that access to improved sanitation facilities and reduced time required to reach drinking water sources are significantly associated with improved nutritional outcomes among children benefiting from the HIMO program. Plausibly, the observed association between improved sanitation and reduced stunting and underweight may operate through reduced exposure to pathogens from unclean environments, with potential benefits for gut function, nutrient absorption, and protection against enteric diseases (78, 79). Evidence from a remote area of Central Madagascar reinforces this mechanism, showing that children affected by intestinal helminthiases and schistosomiasis are significantly more likely to experience stunting, wasting, and undernutrition (80). Because some helminths infect children through oral ingestion or skin penetration, access to clean and safe environments is essential for preventing disease and repeated infections. Climate variability, unsafe environments, poor sanitation, and food and water contamination can exacerbate the proliferation of these parasites in natural settings, while evidenced risk factors include not washing fruits and vegetables before consumption, frequent contact with animals, and working in rice fields (80). Holcomb et al. (81) found that children with access to the pour-flush toilet and septic tank interventions demonstrated improved nutritional and health outcomes compared with those lacking adequate sanitation under the Maputo sanitation (MapSan) trial program in Mozambique. Other researchers, including Spence et al. (82) and Bliznashka et al. (83) analyzed the effects of social and health interventions on child nutrition and diet using mediation frameworks, but identified maternal and other household-level factors as key mediators rather than improved sanitation and water conditions.

Moreover, shorter time to reach drinking water was associated only with child wasting, with no observed relationship for stunting or underweight. Wasting is strongly linked to recent infections such as diarrheal diseases, which can cause rapid weight loss. The association between shorter water-collection time and reduced wasting may reflect lower exposure to water contamination as nearer sources are typically safer (78). It may also indicate time savings for caregivers when water is more accessible (84). This finding is particularly notable in Southern Madagascar, where arid conditions, unsafe water facilities, and chronic water scarcity coincide with the highest national prevalence of wasting (84). Nevertheless, this result is likely context-specific and warrants further investigation to clarify the pathway linking public works, improved water access, and wasting. It also calls for caution, as wasting is highly time-sensitive and the timing of household participation in HIMO relative to data collection is uncertain in this study. Even so, the positive pathways identified between public works, improved WASH conditions, and child nutrition highlight the importance of clean environments for children's growth and support the integration of WASH improvements into social protection policy.

### 4.4 Factors beyond WASH conditions affecting child nutritional status under the HIMO program

Our ability to identify the specific mediating factors linking public works to child nutrition remains limited, whether these pathways operate through sustained improvements in household socioeconomic conditions or program-induced changes in child dietary practices. This limitation is notable given the modest contribution of WASH-related indicators relative to the overall program effects on HAZ, WAZ, and WHZ. Moreover, the impacts of HIMO likely extend beyond the household, with potential inter-household and community-level effects. From this perspective, improving job opportunities, strengthening labor markets, and fostering an enabling macroeconomic environment may also contribute to better child nutrition outcomes (9). In addition to cash or food transfers, program participants may acquire skills relevant to construction and related activities, expanding their future livelihood opportunities.

However, evidence remains scarce regarding the productive value of the public infrastructure created, the spillover effects of these investments, their macroeconomic significance, and their influence on local labor markets, despite attempts to examine these broader impacts (18). Given that public works are typically short-term interventions designed to provide relief during disasters or lean seasons, the durability of household economic gains is also likely limited. Further research is therefore needed to clarify the pathways linking public works programs to child nutrition. Still, the benefits generated by public works programs are substantial, and our findings align with evidence from similar contexts (20, 21, 68, 85, 86). Such initiatives hold particular relevance for Madagascar, where nearly 39% of children experience chronic malnutrition while only 15% have access to basic sanitation services. A cost-effectiveness analysis is therefore essential to assess the overall value of these programs, taking into account both their direct outcomes and their mediation pathways.

### 4.5 Influence of program leakage and targeting imperfections on WASH and child nutritional outcomes

The analysis of the treated and untreated groups' potential treatment effects raised concerns regarding the heterogeneity of the treatment effects. This was particularly evident for improved sanitation, where the ATE was large but the ATET was modest. From a public goods perspective, sanitation facilities built under public-works programs generate substantial community-level spillovers, allowing non-participants to benefit as much as participants (87). This explains the large ATE relative to the ATET. However, from a targeting perspective, this difference may reflect mistargeting and negative selection into treatment. Households that would benefit most from the program are less likely to participate, while those who do participate experience relatively low marginal gains. This interpretation is further supported by the profile of beneficiaries: except for the richest group, differentiation across wealth categories in program participation was low.

This mistargeting is also exacerbated by the endogenous nature of treatment assignment, which produces strong heterogeneity in treatment effects. The findings for the IV “meeting with a social agent” confirmed this pattern: only households with prior access to information or sanitation facilities benefit from the program, while those with potentially higher marginal returns remain excluded. Barriers such as limited information, time constraints, opportunity costs, and social or organizational obstacles may prevent these households from participating (88). Nevertheless, the poor households that cannot participate may still benefit from the publicly built infrastructure and may see improvements in their children's nutritional outcomes, but only if they live close to these facilities. Addressing these barriers is essential to reduce selection bias and ensure equitable program impact. Spatial factors may also contribute to treatment heterogeneity, particularly when public goods are constructed in areas closer to non-participants (89). Scaling up the program while addressing selection biases and prioritizing high-return households can help reduce this heterogeneity.

In contrast, no such heterogeneity was observed for child nutritional outcomes since LATE is quasi-similar to ATE. Untreated households would have likely achieved similar improvements if they had access to the program, suggesting that factors beyond WASH practices play a major role in shaping child nutrition in the region. Heterogenous factors, including income, local markets, socioeconomic conditions, and household behaviors, likely influence child nutrition in Southern Madagascar (90, 91). Inequality, gender differences, and generational biases exacerbate this situation, leaving thousands of children under severe economic and nutritional poverty (84, 92). Addressing those fundamental causes, however, will require more than a safety-net intervention. The trade-offs between participating in the program and accessing its benefits are substantial, particularly when the tasks demand engagement, time, and physical effort that the poorest households struggle to provide (88). Gehrke et al. (18) highlighted the productive effects of public works, offering public health practitioners a new perspective on program implementation that accounted for these trade-offs. With a public work-centered project that prioritizes public infrastructure (47) and assistance relief on its agenda, the local government can achieve multiple public health goals by promoting household welfare, improving child nutrition, and environmental health.

### 4.6 Strengths, limitations and policy implications

Any effort to improve child nutrition therefore requires an integrated policy framework that addresses both nutrition sensitive- and specific- factors within a multisectoral environment (62, 74). Public work initiatives can be an effective instrument to achieve such a goal if implemented correctly; however, they have never received any particular consideration for combatting malnutrition and public health challenges in Madagascar. Despite numerous studies examining the main drivers of malnutrition and assessing the impacts of cash, in-kind, behavioral, or nutrition-support interventions on household and child outcomes in Madagascar, only Razafindrakoto et al. (49) and later Andrianjaka (13) analyzed the effects of the HIMO program on macroeconomic indicators. Their findings showed that HIMO generated household income benefits, increased consumption, and created added value with positive implications for the economic balance and the labor market. This study complements these contributions by establishing, for the first time, a public-health-centered relationship between public works participation and improved child *Z*-scores. Nevertheless, the short-term nature of opportunities and benefits provided under public works schemes underscores the need for policy measures that support the expansion and institutionalization of these programs. Strengthening technical and institutional capacity, ensuring timely implementation, and improving information flows are also essential for maximizing their contribution to sustainable development goals.

This study's results must be interpreted with caution. First, this study relied on cross-sectional data, which inherently limits the ability to establish temporal ordering and confirm causal directionality. As a result, the analysis primarily reflects an assessment of associations between project participation and potential outcomes rather than definitive causal effects. Given the weakness of the treatment IV, the direct effects mentioned in this study can be theoretically limited, and other survey methodologies, including randomized control trials, are suggested to fully capture the effects of WASH mediators on child outcomes. Second, constraints in the available information concerning the project design, main targets, and implementation process prevent us from capturing the extent of the effects of the HIMO program on households' WASH practices and child nutrition. Third, the potential bias in the estimation of the indirect effect analysis can exist since we neither specified the determinants of the outcomes, nor those of the mediators. Fourth, the program coverage remains low (nearly 16%) despite the generalized poverty level in the zone. Such inconvenience may prevent us from succinctly capturing the extent of the program's effects on households and children's outcomes. Lastly, the research was limited to facility-based rather than behavioral WASH outcomes because of the limitations in the dataset. The consideration of household behaviors in the use of these facilities along with their management is key in unraveling the pathway from public works to public health.

Nevertheless, this study has strong policy implications regarding the possible impact of public works programs on welfare poverty alleviation and improving children's nutritional status. First, we suggest that complementary measures should accompany these projects after their implementation to ease the use of the newly built infrastructure by the local community. Second, a behavioral nudge complementary package might be necessary to induce the multi-potential of these programs in improving developmental outcomes at the individual and household levels. Additionally, these supplementary components can also be efficient in improving stunting among children. Third, the intra-household dynamics in terms of economic benefits appear to be consistent with our findings. Nutrition-sensitive social protection programs that support the socioeconomic condition of the household, community infrastructure, and child nutrition can be effective in improving household and child welfare in Southern Madagascar and protect the livelihood during shocks. Otherwise, promoting the economic side of these projects to cover not only a post-disaster period or lean season but in a longer term would be importantly beneficial to livelihood. The possibility of extending these projects into a more formal employment opportunity can also be suggested to sustain economic growth and nutrition.

## 5 Conclusion and recommendations

This study examined the direct and mediator-adjusted associations of HIMO participation with child nutrition, through the potential mediating role of WASH-related variables. In comparison with other studies investigating WASH practices and child nutrition (23), this study evaluated whether participation in these programs enhanced children's access to improved sanitation and drinking-water source facilities and reduced the time required to access the nearest drinking-water source. Second, it assessed the association between program participation and children's nutritional outcomes, including HAZ, WAZ, and WHZ. Lastly, it explored the pathway from public-works participation to child nutrition by including the respective WASH variables as mediators. Evidence from this study indicated that public works are likely effective in addressing child nutrition and breaking the barriers to accessing basic infrastructure for households in Southern Madagascar. However, the mediating effects of WASH practices were relatively low, especially for stunting, highlighting the importance of other factors, such as socioeconomic, behavioral, and environmental factors, in shaping child nutrition in such a vulnerable context. The burden of economic poverty was also high, and it hindered efforts to address malnutrition and household welfare. Key recommendations include improving the program targeting and delivery approaches, addressing constraints and barriers to program participation, and scaling up projects to include mediator potentials alongside wealth and nutritional-based outcomes. A pioneering aspect of this research was that it established mediation pathways linking public works to child nutrition through the intermediary effects of WASH practices in Madagascar. It also stood out from other studies by addressing both treatment and mediator endogeneity in its framework.

## Data Availability

Publicly available datasets were analyzed in this study. This data can be found here: Repository 1: Name: UNICEF Multiple Indicator Cluster Surveys (MICS), Link: https://mics.unicef.org/surveys Accession number: Madagascar MICS6 (2018) Repository 2: Name: DHS Program (Demographic and Health Surveys), Link: https://dhsprogram.com/data/available-datasets.cfm Accession number: Madagascar DHS 2021 Spatial Covariates.
