# Supplementary material for: Assessing the associations between public works participation, household water and sanitation conditions, and child nutrition in Southern Madagascar: a mediation analysis
Source: Front Public Health. 2026 Jun 22;14:1846565. doi: 10.3389/fpubh.2026.1846565 (PMC13333734; doi:10.3389/fpubh.2026.1846565)
Supplement: Supplementary file 1 [file Table_1.docx]

Supplementary Material

# Potential outcome framework

## Direct effect analysis

The direct effect analysis consists of evaluating the direct effect of programme participation on household and child outcome variables. We followed the potential outcome framework for all analyses. The average treatment effect (ATE), potential outcome means (POM), and average treatment of the treated (ATET) are given by the following equations:


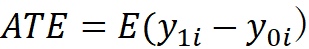

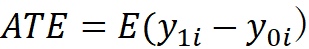
, (1)


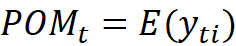

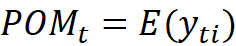
, (2)


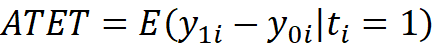

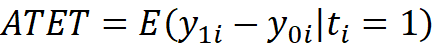
, (3)

where
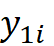

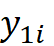
is the potential outcome of receiving treatment, i represents the observation unit i.e. household or child,
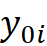

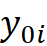
 is the potential outcome when treatment is not received,
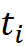

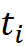
 is the observed binary treatment,
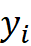

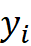
 is the observed outcome.
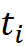

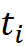
 is conditional on a set of regressors
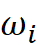

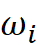
 that does not need to differ from
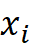

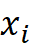
, the regressors of the outcome
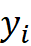

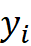
:


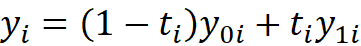

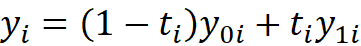
, (4)


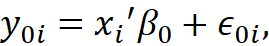

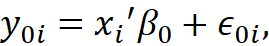
 (5)


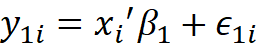

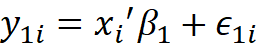
, (6)


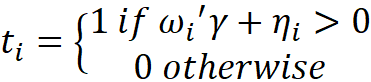

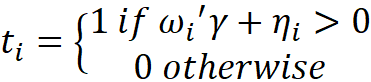
. (7)

In our model,
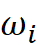

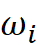
 and
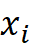

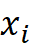
 are same as we intend to directly assess the impact of the programme on the outcome variables and they represent the set of regressors that fully predict the treatment variable. They include dummies for wealth ranking groups, religious affiliation, regional distribution, ITN possession, and gender of the household head. β are γ are the respective coefficients vectors of
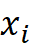

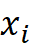
 and
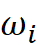

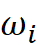
,
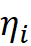

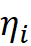
 and
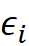

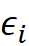
 are error terms.

When the treatment is endogenous, it requests the use of an instrumental variable-based technique to estimate the reduced form of the outcome function [26]. (7) becomes:


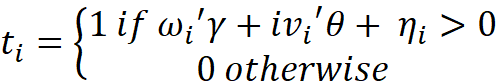

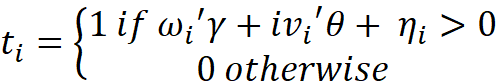
. (8)

Where *iv* is the instrument for the treatment variable.

## Indirect effect analysis

We assessed the ATE and ATET of the workfare programme based on the previous approach but incorporated each WASH variable that was significantly impacted by the programme as a regressor into the outcome model. Before assessing the model, we subjected each WASH-related variable to an endogenous test to verify the correlation between the outcome and the mediator model's error terms,. As a result of endogeneity, we instrumented the mediator with a spatial covariate that was neither related to the outcome nor the treatment.


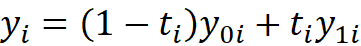

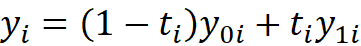
, (10)


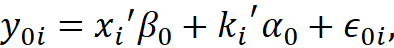

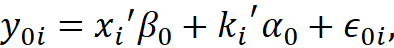
 (11)


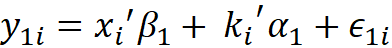

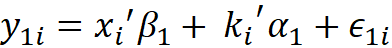
, (12)


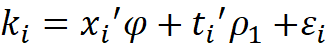

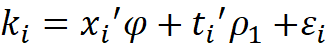
 if
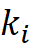

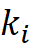
 is exogenous (13)


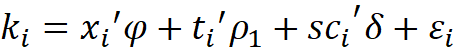

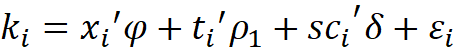
 if
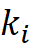

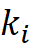
 is endogenous (14)


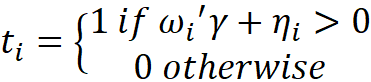

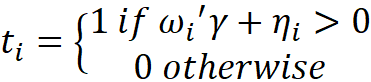
 if
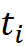

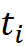
 is exogenous (15)


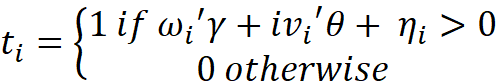

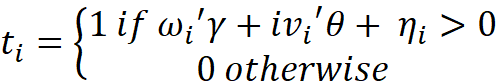
 if
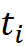

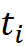
 is endogenous (16)

Where
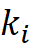

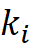
 represents a WASH variable,
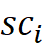

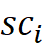
 is a spatial covariate and instrument for
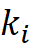

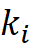
, and
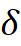

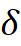
 is the coefficient vector of
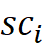

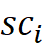
.
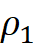

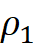
 represents the coefficient of the treatment
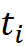

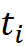
 in the mediation equation.

# Supplementary Tables

Table SI: Estimated coefficients from the direct effect analysis of program participation on household WASH practices

| **Variables** | **Improved sanitation** | **Time access to drinking-water** | **Improved water-sources ^α^** |
| --- | --- | --- | --- |
| **Potential outcomes equation (Treatment with control variables)** |  |  |  |
| **Religion** |  |  |  |
| No religion x without programme | base | base | base |
| No religion x with programme | base | base | base |
| Christianism x without programme | 0.120 | -0.100 | 0.238 |
| Christianism x with programme | -0.240 | -0.364** | 0.034 |
| Other religions x without programme | 0.262 | -0.123 | 0.337* |
| Other religions x with programme | -0.172 | -0.437** | -0.467 |
| **Economic status** |  |  |  |
| Poorest x without programme | base | base | base |
| Poorest x with programme | base | base | base |
| Lower-middle poor x without programme | 0.536*** | 0.114 | 0.211* |
| Lower-middle poor x with programme | 0.350 | 0.347** | 0.277 |
| Middle poor x without programme | 0.751*** | 0.175 | 0.430*** |
| Middle poor x with programme | 0.658* | 0.248 | 0.719** |
| Upper-rich x without programme | 1.170*** | 0.146 | 1.255*** |
| Upper-rich x with programme | 0.647** | 0.103 | 1.089*** |
| Richest x without programme | 2.015*** | 0.128 | 1.815*** |
| Richest x with programme | 1.739*** | 1.032** | 3.529*** |
| **Regions** |  |  |  |
| Haute Matsiatra x without programme | base | base | base |
| Haute Matsiatra x with programme | base | base | base |
| Amoron’I Mania x without programme | 0.083 | 0.494** | -0.696** |
| Amoron’I Mania x with programme | 0.474 | 0.511 | -0.349 |
| Vatovavy Fitovinany x without programme | -0.104 | 0.157 | -0.635** |
| Vatovavy Fitovinany x with programme | 0.184 | 0.039 | 0.516 |
| Ihorombe x without programme | -0.863*** | 0.266 | -0.507* |
| Ihorombe x with programme | -0.233 | 0.434 | 0.073 |
| Atsimo Atsinanana x without programme | -0.779** | -0.134 | -0.726** |
| Atsimo Atsinanana x with programme | -0.078 | -0.335 | -0.124 |
| Atsimo Andrefana x without programme | 0.142 | -0.563** | -0.296 |
| Atsimo Andrefana x with programme | -0.044 | -0.890*** | 0.868 |
| Androy x without programme | -0.524* | -0.903*** | 0.133 |
| Androy x with programme | -0.515* | -0.921*** | 0.761 |
| Anosy x without programme | -0.386 | -0.371 | -0.167 |
| Anosy x with programme | -0.396 | -0.304 | -0.246 |
| Menabe x without programme | -1.110*** | -0.123 | -0.162 |
| Menabe x with programme | -0.110 | -0.247 | 0.610 |
| **Head of the household** |  |  |  |
| Male x without programme | base | base | base |
| Male x with programme | base | base | base |
| Female x without programme | -0.110 | 0.166* | 0.237* |
| Female x with programme | 0.151*** | 0.292* | 0.211 |
| **Insecticide-treated nets possession** |  |  |  |
| Without ITNx without programme | base | base | base |
| Without ITNx with programme | base | base | base |
| With ITNx without programme | -0.070 | -0.151 | 0.234* |
| With ITNx with programme | -0.025 | -0.030 | 0.211 |
| **Programme participation** |  |  |  |
| Without programme | -1.584*** | 0.323 | -1.109*** |
| With programme | 0.345 | 1.913*** | -1.125*** |
| **Meeting social agent** (ref: No) |  |  | 0.103 |
| Yes |  |  | -0.068 |
| **Treatment equation (with control variables and instrumental variable)** | | | |
| **Religion** (ref: No religion) |  |  |  |
| Christianism | 0.023* | 0.292** |  |
| Other religions | 0.161 | 0.239* |  |
| **Economic status** (ref: Poorest) |  |  |  |
| Lower-middle poor | 0.006 | 0.054 |  |
| Middle poor | -0.044 | -0.029 |  |
| Upper-rich | 0.067 | 0.046 |  |
| Richest | -0.906*** | -0.876*** |  |
| **Regions** (ref: Haute Matsiatra) |  |  |  |
| Amoron’I Mania | -0.368 | -0.368 |  |
| Vatovavy Fitovinany | -0.064 | 0.004 |  |
| Ihorombe | -0.611* | -0.633* |  |
| Atsimo Atsinanana | -0.189 | -0.153 |  |
| Atsimo Andrefana | 0.215 | 0.211 |  |
| Androy | 0.156 | 0.158 |  |
| Anosy | -0.214 | -0.178 |  |
| Menabe | -0.499 | -0.468 |  |
| **Head of househol**d (ref: Male) |  |  |  |
| Female | -0.207* | -0.211* |  |
| **Insecticide-Treated Nets possession** (ref: No) |  |  |  |
| Yes | 0.262 | 0.235 |  |
| **Meeting social agent** ^b^ (ref: No) |  |  |  |
| Yes | 0.272 *** | 0.163* |  |
| **corr(e.HIMO,e.outcome)** | -0.868*** | -0.985*** |  |
| **Constant** | -1.202*** | -1.218*** |  |
| **Observations** | **5879** | **5879** | **5879** |

*** p<0.001, ** p<0.01, * p<0.05; α: exogenous treatment; b: instrument variable for the treatment equation

Table SII: Estimated coefficients from the direct effect analysis of program participation on child nutrition

| **Variables** | **HAZ** | **WAZ** | **WHZ** |
| --- | --- | --- | --- |
| **Potential outcomes equation (Treatment with control variables) Obs: 5879** | | | |
| **Religion** |  |  |  |
| No religion x without program | base | base | base |
| No religion x with program | base | base | base |
| Christianism x without program | -0.031 | -0.003 | -0.010 |
| Christianism x with program | -0.197 | -0.186 | -0.176 |
| Other religions x without program | -0.055 | -0.060 | -0.041 |
| Other religions x with program | -0.0418 | -0.191 | -0.327** |
| **Economic status** |  |  |  |
| Poorest x without program | base | base | base |
| Poorest x with program | base | base | base |
| Lower-middle poor x without program | -0.073 | -0.018 | 0.029 |
| Lower-middle poor x with program | 0.002 | 0.043 | 0.049 |
| Middle poor x without program | -0.104 | -0.018 | 0.077 |
| Middle poor x with program | 0.155 | 0.139 | 0.0542 |
| Upper-rich x without program | -0.063 | 0.061 | 0.111 |
| Upper-rich x with program | 0.028 | 0.169 | 0.167 |
| Richest x without program | 0.500*** | 0.471*** | 0.380*** |
| Richest x with program | 1.149*** | 1.107*** | 0.925*** |
| **Regions** |  |  |  |
| Haute Matsiatra x without program | base | base | base |
| Haute Matsiatra x with program | base | base | base |
| Amoron’I Mania x without program | 0.0480 | -0.139 | -0.156 |
| Amoron’I Mania x with program | 0.349 | -0.0154 | -0.181 |
| Vatovavy Fitovinany x without program | 0.220 | -0.131 | -0.367** |
| Vatovavy Fitovinany x with program | 0.429 | 0.125 | -0.121 |
| Ihorombe x without program | 0.875*** | 0.532*** | 0.133 |
| Ihorombe x with program | 1.470*** | 0.805*** | 0.274 |
| Atsimo Atsinanana x without program | 1.337*** | 0.944*** | 0.336** |
| Atsimo Atsinanana x with program | 1.313*** | 1.065*** | 0.542** |
| Atsimo Andrefana x without program | 0.257* | 0.024 | -0.213* |
| Atsimo Andrefana x with program | 0.481* | 0.135 | -0.279 |
| Androy x without program | 0.443** | 0.130 | -0.220* |
| Androy x with program | 0.557* | 0.284* | -0.101 |
| Anosy x without program | 0.490*** | 0.050 | -0.288** |
| Anosy x with program | 0.869*** | 0.417* | -0.110 |
| Menabe x without program | 0.731*** | 0.222* | -0.205 |
| Menabe x with program | 0.536 | 0.080 | -0.084 |
| **Head of the household** |  |  |  |
| Male x without program | base | base | base |
| Male x with program | base | base | base |
| Female x without program | -0.104 | -0.107 | -0.034 |
| Female x with program | 0.166 | 0.127 | 0.146 |
| **Insecticide-treated nets possession** |  |  |  |
| Without ITNx without program | base | base | base |
| Without ITNx with program | base | base | base |
| With ITNx without program | 0.005 | -0.0105 | -0.082 |
| With ITNx with program | -0.275 | -0.247 | -0.302* |
| **Program participation** |  |  |  |
| Without program | -2.260*** | -1.591*** | -0.521*** |
| With program | -0.854* | -0.704** | 0.506* |
| **Treatment equation (with control variables and instrumental variable)** | | | |
| **Religion** (ref: No religion) |  |  |  |
| Christianism | 0.227* | 0.222* | 0.226* |
| Other religions | 0.161 | 0.148 | 0.149 |
| **Economic status** (ref: Poorest) |  |  |  |
| Lower-middle poor | 0.016 | 0.008 | -0.001 |
| Middle poor | -0.049 | -0.057 | -0.057 |
| Upper-rich | 0.077 | 0.063 | 0.051 |
| Richest | -0.842*** | -0.880*** | -0.896*** |
| **Regions** (ref: Haute Matsiatra) |  |  |  |
| Amoron’I Mania | -0.362 | -0.371 | -0.391* |
| Vatovavy Fitovinany | -0.065 | -0.0834 | -0.099 |
| Ihorombe | -0.614* | -0.605** | -0.619* |
| Atsimo Atsinanana | -0.206 | -0.202 | -0.202 |
| Atsimo Andrefana | 0.194 | 0.196 | 0.186 |
| Androy | 0.151 | 0.161 | 0.153 |
| Anosy | -0.241 | -0.250 | -0.249 |
| Menabe | -0.498* | -0.496 | -0.504 |
| **Head of household** (ref: Male) |  |  |  |
| Female | -0.201* | -0.209* | -0.214* |
| **Insecticide-Treated Nets possession** (ref: No) |  |  |  |
| Yes | 0.268 | 0.253 | 0.254 |
| **Meeting social agent** ^b^ (ref: No) |  |  |  |
| Yes | 0.266*** | 0.298*** | 0.285*** |
| **var(e.outcome)** | 2.008 | 1.198 | 1.124 |
| **corr(e.HIMO,e.outcome)** | -0.537*** | -0.430*** | -0.472*** |
| **Constant** | -1.197*** | -1.181*** | -1.168*** |

*** p<0.001, ** p<0.01, * p<0.05; b: instrument variable for the treatment equation

Table SIII: Estimated coefficients from the indirect effect analysis of program participation on child nutrition through the mediating effect of the access to improved sanitation

| **Variables** | **HAZ** | **Sanitation** | **WAZ** | **Sanitation** | **WHZ** | **Sanitation ^γ^** |
| --- | --- | --- | --- | --- | --- | --- |
|  | **(outcome)** | **(mediator)** | **(outcome)** | **(mediator)** | **(outcome)** | **(mediator)** |
| **Potential outcomes equation (Treatment with mediator and control variables) Obs: 5879** | | | | | | |
| **Religion** |  |  |  |  |  |  |
| No religion x without program | base |  | base |  | base |  |
| No religion x with program | base |  | base |  | base |  |
| Christianism x without program | -0.049 |  | -0.014 |  | -0.010 |  |
| Christianism x with program | -0.205 |  | -0.203* |  | -0.182 |  |
| Other religions x without program | -0.094 |  | -0.083 |  | -0.041 |  |
| Other religions x with program | -0.089 |  | -0.227 |  | -0.328** |  |
| **Economic status** |  |  |  |  |  |  |
| Poorest x without program | base |  | base |  | base |  |
| Poorest x with program | base |  | base |  | base |  |
| Lower-middle poor x without program | -0.182* |  | -0.081 |  | 0.030 |  |
| Lower-middle poor x with program | -0.113 |  | -0.011 |  | 0.070 |  |
| Middle poor x without program | -0.299** |  | -0.129 |  | 0.080 |  |
| Middle poor x with program | -0.018 |  | 0.071 |  | 0.098 |  |
| Upper-rich x without program | -0.437** |  | -0.153 |  | 0.116 |  |
| Upper-rich x with program | -0.281 |  | 0.020 |  | 0.216 |  |
| Richest x without program | -0.256 |  | 0.041 |  | 0.394*** |  |
| Richest x with program | 0.395 |  | 0.774* |  | 1.031*** |  |
| **Regions** |  |  |  |  |  |  |
| Haute Matsiatra x without program | base |  | base |  | base |  |
| Haute Matsiatra x with program | base |  | base |  | base |  |
| Amoron’I Mania x without program | 0.002 |  | -0.163* |  | -0.154 |  |
| Amoron’I Mania x with program | 0.312 |  | -0.013 |  | -0.151 |  |
| Vatovavy Fitovinany x without program | 0.239 |  | -0.120 |  | -0.368** |  |
| Vatovavy Fitovinany x with program | 0.472* |  | 0.160 |  | -0.107 |  |
| Ihorombe x without program | 1.071*** |  | 0.647*** |  | 0.132 |  |
| Ihorombe x with program | 1.594*** |  | 0.867*** |  | 0.242 |  |
| Atsimo Atsinanana x without program | 1.514*** |  | 1.046*** |  | 0.333** |  |
| Atsimo Atsinanana x with program | 1.471*** |  | 1.147*** |  | 0.526** |  |
| Atsimo Andrefana x without program | 0.214 |  | -0.001 |  | -0.213* |  |
| Atsimo Andrefana x with program | 0.479* |  | 0.128 |  | -0.274 |  |
| Androy x without program | 0.611*** |  | 0.222* |  | -0.223 |  |
| Androy x with program | 0.687** |  | 0.344* |  | -0.127 |  |
| Anosy x without program | 0.620*** |  | 0.125 |  | -0.289** |  |
| Anosy x with program | 0.988*** |  | 0.472** |  | -0.136 |  |
| Menabe x without program | 0.948*** |  | 0.349** |  | -0.207 |  |
| Menabe x with program | 0.721 |  | 0.181 |  | -0.103 |  |
| **Head of the household** |  |  |  |  |  |  |
| Male x without program | base |  | base |  | base |  |
| Male x with program | base |  | base |  | base |  |
| Female x without program | -0.112 |  | -0.111 |  | -0.033 |  |
| Female x with program | 0.138 |  | 0.140 |  | 0.172 |  |
| **Insecticide-treated nets possession** |  |  |  |  |  |  |
| Without ITNx without program | base |  | base |  | base |  |
| Without ITNx with program | base |  | base |  | base |  |
| With ITNx without program | 0.007 |  | -0.012 |  | -0.083 |  |
| With ITNx with program | -0.269 |  | -0.238 |  | -0.298* |  |
| **Improved sanitation** |  |  |  |  |  |  |
| Unimproved sanitation x without program | base |  | base |  | base |  |
| Unimproved sanitation x with program | base |  | base |  | base |  |
| Improved sanitation x without program | 1.340*** |  | 0.767** |  | -0.020 |  |
| Improved sanitation x with program | 1.068*** |  | 0.464 |  | -0.202* |  |
| **Program participation** |  |  |  |  |  |  |
| Without program | -2.378*** |  | -1.663*** |  | -0.520*** |  |
| With program | -1.050* |  | -0777* |  | 0.531 |  |
| **Treatment/Mediator equation (with control variables and instrumental variable)** | | | | | | |
| **Religion** (ref: No religion) |  |  |  |  |  |  |
| Christianism | 0.225* | 0.142 | 0.221* | 0.122 | 0.227** |  |
| Other religions | 0.159 | 0.255 | 0.148 | 0.235 | 0.148 |  |
| **Economic status** (ref: Poorest) |  |  |  |  |  |  |
| Lower-middle poor | 0.010 | 0.561*** | 0.006 | 0.576*** | -0.001 |  |
| Middle poor | -0.061 | 0.821*** | -0.062 | 0.843*** | -0.057 |  |
| Upper-rich | 0.067 | 1.204*** | 0.060 | 1.208*** | 0.053 |  |
| Richest | -0.860*** | 1.823*** | -0.886*** | 1.896*** | -0.896*** |  |
| **Regions** (ref: Haute Matsiatra) |  |  |  |  |  |  |
| Amoron’i Mania | -0.373 | 0.017 | -0.373 | -0.067 | -0.391 |  |
| Vatovavy Fitovinany | -0.074 | 0.003 | -0.086 | -0.004 | -0.099 |  |
| Ihorombe | -0.605* | -0.958*** | -0.604* | -0.974*** | -0.619*** |  |
| Atsimo Atsinanana | -0.202 | -0.874*** | -0.202 | -0.846** | -0.202 |  |
| Atsimo Andrefana | 0.191 | 0.205 | 0.195 | 0.215 | 0.187 |  |
| Androy | -0.155 | -0.488* | 0.160 | -0.496* | 0.152 |  |
| Anosy | -0.244 | -0.509 | -0.253 | -0.544* | -0.248 |  |
| Menabe | -0.495 | -1.179*** | -0.497 | -1.208*** | -0.504 |  |
| **Head of household** (ref: Male) |  |  |  |  |  |  |
| Female | -0.204* | 0.075 | -0.210* | 0.093 | -0.213* |  |
| **Insecticide-Treated Nets possession** (ref: No) |  |  |  |  |  |  |
| Yes | 0.259 | 0.011* | 0.251 | 0.254 | 0.254 |  |
| **Meeting social agent** ^b^ (ref: No) |  |  |  |  |  |  |
| Yes | 0.273*** |  | 0.299*** |  | 0.285*** |  |
| **Housing status** ^c^ (ref: Tenant) |  |  |  |  |  |  |
| Homeowner |  | -0.356** |  | -0.393** |  |  |
| **var(e.outcome)** | 2.157 |  | 1.381 |  | 1.127 |  |
| **corr(e.HIMO, e.outcome)** | -0.481** |  | -0.421*** |  | -0.479*** |  |
| **corr(e. impsanit, e.outcome)** |  | -0.549*** |  | -0.392*** |  |  |
| **corr(e.impsanit, e.HIMO)** |  | 0.185 |  | 0.454 |  |  |
| **Constant** | -1.183*** | -1.307** | -1.176*** | -1.192*** | -1.169*** |  |

*** p<0.001, ** p<0.01, * p<0.05; γ: exogenous mediator**;** b: IV for the treatment equation; c: IV for the mediator

Table SIV: Estimated coefficients from the indirect effect analysis of program participation on child nutrition through the mediating effect of time to access to drinking-water sources

| **Variables** | **HAZ** | **Time to water ^γ^** | **WAZ** | **Time to water ^γ^** | **WHZ** | **Time to water** |
| --- | --- | --- | --- | --- | --- | --- |
|  | **(outcome)** | **(mediator)** | **(outcome)** | **(mediator)** | **(outcome)** | **(mediator)** |
| **Potential outcomes equation (Treatment with mediator and control variables) Obs: 5879** | | | | | | |
| **Religion** |  |  |  |  |  |  |
| No religion x without program | base |  | base |  | base |  |
| No religion x with program | base |  | base |  | base |  |
| Christianism x without program | -0.031 |  | -0.003 |  | -0.002 |  |
| Christianism x with program | -0.196 |  | -0.187 |  | -0.158 |  |
| Other religions x without program | -0.055 |  | -0.061 |  | -0.0001 |  |
| Other religions x with program | -0.040 |  | -0.194 |  | -0.291* |  |
| **Economic status** |  |  |  |  |  |  |
| Poorest x without program | base |  | base |  | base |  |
| Poorest x with program | base |  | base |  | base |  |
| Lower-middle poor x without program | -0.071 |  | -0.016 |  | -0.061 |  |
| Lower-middle poor x with program | -0.001 |  | 0.048 |  | 0.030 |  |
| Middle poor x without program | -0.102 |  | -0.015 |  | -0.164 |  |
| Middle poor x with program | 0.153 |  | 0.142 |  | -0.031 |  |
| Upper-rich x without program | -0.060 |  | 0.064 |  | 0.001 |  |
| Upper-rich x with program | -0.026 |  | 0.172 |  | 0.094 |  |
| Richest x without program | 0.498** |  | 0.468*** |  | 0.433*** |  |
| Richest x with program | 1.146*** |  | 1.110*** |  | 0.929*** |  |
| **Regions** |  |  |  |  |  |  |
| Haute Matsiatra x without program | base |  | base |  | base |  |
| Haute Matsiatra x with program | base |  | base |  | base |  |
| Amoron’I Mania x without program | 0.052 |  | -0.134 |  | -0.253 |  |
| Amoron’I Mania x with program | 0.348 |  | -0.014 |  | -0.280 |  |
| Vatovavy Fitovinany x without program | 0.223 |  | -0.127 |  | -0.439** |  |
| Vatovavy Fitovinany x with program | 0.428 |  | 0.126 |  | -0.169 |  |
| Ihorombe x without program | 0.875*** |  | 0.532*** |  | 0.164 |  |
| Ihorombe x with program | 1.470*** |  | 0.805*** |  | 0.253 |  |
| Atsimo Atsinanana x without program | 1.333*** |  | 0.939*** |  | 0.484** |  |
| Atsimo Atsinanana x with program | 1.316*** |  | 1.060*** |  | 0.669 |  |
| Atsimo Andrefana x without program | 0.248 |  | 0.014 |  | 0.066 |  |
| Atsimo Andrefana x with program | 0.487* |  | 0.127 |  | 0.017 |  |
| Androy x without program | 0.428** |  | 0.114 |  | 0.198 |  |
| Androy x with program | 0.565* |  | 0.274 |  | 0.311 |  |
| Anosy x without program | 0.482*** |  | 0.042 |  | -0.064 |  |
| Anosy x with program | 0.871*** |  | 0.414* |  | 0.043 |  |
| Menabe x without program | 0.726*** |  | 0.216* |  | 0.001 |  |
| Menabe x with program | 0.541 |  | 0.072 |  | 0.052 |  |
| **Head of the household** |  |  |  |  |  |  |
| Male x without program | base |  | base |  | base |  |
| Male x with program | base |  | base |  | base |  |
| Female x without program | -0.103 |  | 0.069 |  | -0.065 |  |
| Female x with program | 0.165 |  | 0.129 |  | 0.096 |  |
| **Insecticide-treated nets possession** |  |  |  |  |  |  |
| Without ITNx without program | base |  | base |  | base |  |
| Without ITNx with program | base |  | base |  | base |  |
| With ITNx without program | 0.004 |  | -0.011 |  | -0.101 |  |
| With ITNx with program | -0.276 |  | -0.245 |  | -0.276* |  |
| **Time to access drinking water-sources** |  |  |  |  |  |  |
| More time x without program | base |  | base |  | base |  |
| More time x with program | base |  | base |  | base |  |
| Less time x without program | -0.036 |  | -0.039** |  | 1.021*** |  |
| Less time x with program | 0.013 |  | -0.021*** |  | 0.852*** |  |
| **Program participation** |  |  |  |  |  |  |
| Without program | -2.233*** |  | -1.562*** |  | -1.273*** |  |
| With program | -0.864* |  | -0.692* |  | -0.012 |  |
| **Treatment/Mediator equation (with control variables and instrumental variable)** | | | | | | |
| **Religion** (ref: No religion) |  |  |  |  |  |  |
| Christianism | 0.227* |  | 0.222* |  | 0.253* | 0.023 |
| Other religions | 0.161 |  | 0.148 |  | 0.178 | -0.071 |
| **Economic status** (ref: Poorest) |  |  |  |  |  |  |
| Lower-middle poor | 0.016 |  | 0.008 |  | -0.033 | 0.232** |
| Middle poor | -0.049 |  | -0.058 |  | -0.083 | 0.227* |
| Upper-rich | 0.077 |  | 0.063 |  | -0.004 | 0.253** |
| Richest | -0.841*** |  | -0.880*** |  | -0.995*** | -0.273 |
| **Regions** (ref: Haute Matsiatra) |  |  |  |  |  |  |
| Amoron’i Mania | -0.362 |  | -0.370 |  | -0.393 | 0.297 |
| Vatovavy Fitovinany | -0.066 |  | -0.083 |  | -0.082 | -0.071 |
| Ihorombe | -0.614* |  | -0.605* |  | -0.589 | -0.334 |
| Atsimo Atsinanana | 0.206 |  | -0.202 |  | -0.182 | -0.604*** |
| Atsimo Andrefana | 0.193 |  | 0.196 |  | 0.207 | -0.556** |
| Androy | 0.151 |  | 0.160 |  | 0.170 | -0.790** |
| Anosy | -0.241 |  | -0.250 |  | -0.222 | -0.528** |
| Menabe | -0.498 |  | -0.496 |  | -0.453 | -0.603** |
| **Head of household** (ref: Male) |  |  |  |  |  |  |
| Female | -0.201* |  | -0.209* |  | -0.197* | 0.037 |
| **Insecticide-Treated Nets possession** (ref: No) |  |  |  |  |  |  |
| Yes | 0.268 |  | 0.252 |  | 0.219 | 0.063 |
| **Meeting social agent** ^b^ (ref: No) |  |  |  |  |  |  |
| Yes | 0.266*** |  | 0.298*** |  | 0.234** |  |
| **Enhanced vegetation index** ^c^ |  |  |  |  |  | 2.271** |
| **var(e.outcome)** | 2.007 |  | 1.197 |  | 1.384 |  |
| **corr(e.HIMO, e.outcome)** | -0.537*** |  | -0.429*** |  | -0.505*** |  |
| **corr(e. impsanit, e.outcome)** |  |  |  |  |  | -0.624*** |
| **corr(e.impsanit, e.HIMO)** |  |  |  |  |  | 0.730** |
| **Constant** | -1.197*** |  | -1.181*** |  | -1.142*** | -1.232** |

*** p<0.001, ** p<0.01, * p<0.05; γ: exogenous mediator**;** b: IV for the treatment equation; c: IV for the mediator
